# Glyoxalase 1 Prevents Chronic Hyperglycemia Induced Heart-Explant Derived Cell Dysfunction

**DOI:** 10.7150/thno.36639

**Published:** 2019-08-09

**Authors:** Melanie Villanueva, Connor Michie, Sandrine Parent, Georges N Kanaan, Ghazaleh Rafatian, Pushpinder Kanda, Bin Ye, Wenbin Liang, Mary-Ellen Harper, Darryl R Davis

**Affiliations:** 1 University of Ottawa Heart Institute, Division of Cardiology, Department of Medicine, University of Ottawa, Ottawa, Canada K1Y4W7; 2 Department of Biochemistry, Microbiology and Immunology, Faculty of Medicine, University of Ottawa, Ottawa, Canada K1H8M5; 3 Department of Cellular and Molecular Medicine, Faculty of Medicine, University of Ottawa, Ottawa, Canada K1H8M5

**Keywords:** cardiac stem cells, diabetes, extracellular vesicles, hyperglycemia, heart failure, myocardial infarction, oxidative stress, reactive dicarbonyls

## Abstract

Decades of work have shown that diabetes increases the risk of heart disease and worsens clinical outcomes after myocardial infarction. Because diabetes is an absolute contraindication to heart transplant, cell therapy is increasingly being explored as a means of improving heart function for these patients with very few other options. Given that hyperglycemia promotes the generation of toxic metabolites, the influence of the key detoxification enzyme glyoxalase 1 (Glo1) on chronic hyperglycemia induced heart explant-derived cell (EDC) dysfunction was investigated.

**Methods:** EDCs were cultured from wild type C57Bl/6 or Glo1 over-expressing transgenic mice 2 months after treatment with the pancreatic beta cell toxin streptozotocin or vehicle. The effects of Glo1 overexpression was evaluated using *in vitro* and *in vivo* models of myocardial ischemia.

**Results:** Chronic hyperglycemia reduced overall culture yields and increased the reactive dicarbonyl cell burden within EDCs. These intrinsic cell changes reduced the angiogenic potential and production of pro-healing exosomes while promoting senescence and slowing proliferation. Compared to intra-myocardial injection of normoglycemic cells, chronic hyperglycemia attenuated cell-mediated improvements in myocardial function and reduced the ability of transplanted cells to promote new blood vessel and cardiomyocyte growth. In contrast, Glo1 overexpression decreased oxidative damage while restoring both cell culture yields and EDC-mediated repair of ischemic myocardium. The latter was associated with enhanced production of pro-healing extracellular vesicles by Glo1 cells without altering the pro-healing microRNA cargo within.

**Conclusions:** Chronic hyperglycemia decreases the regenerative performance of EDCs. Overexpression of Glo1 reduces dicarbonyl stress and prevents chronic hyperglycemia-induced dysfunction by rejuvenating the production of pro-healing extracellular vesicles.

## Introduction

Over the past decade, heart-derived cell therapy has emerged as a promising means to promote therapeutic regeneration 1-4 but successful translation of autologous therapeutics may ultimately be frustrated by the adverse influence of medical comorbidities on the regenerative performance of transplanted cells 5-7. Previously, we have shown that chronic hyperglycemia reduces the ability of heart explant-derived cells (EDCs) to repair injured myocardium 5. EDCs originate exclusively from cultured heart tissue with no detectable seeding by extra-cardiac sources 8. Similar to other mesenchymal cell products, EDCs and their expanded progeny (cardiosphere-derived cells) uniformly express transforming growth factor receptor beta receptor (aka endoglin or CD105) 9, 10. Therapeutic efficacy is difficult to predict using molecular profiling alone but appears to be largely mediated by the paracrine stimulation of endogenous repair by the active CD90- fraction 11. Despite this progress, the fundamental mechanisms underlying the adverse effects of hyperglycemia are unclear but may be related to accumulation of methylglyoxal which limits transplanted cell stimulation of angiogenesis 5. Somatic gene transfer of the key detoxification enzyme glyoxalase 1 (Glo1) restored the proangiogenic capacity of diabetic EDCs suggesting a means of reversing diabetic cell dysfunction by interfering with the accumulation of reactive dicarbonyls. Interestingly, these changes occurred despite negligible effects on both the paracrine cytokine signature and survival of transplanted cells; suggesting that the fundamental changes wrought by chronic hyperglycemia must alter other cell secreted factors that promote endogenous cardiac repair (such as extracellular vesicles, EVs) 12, 13.

In this study, we use a transgenic murine model of Glo1 overexpression to critically evaluate the effect of chronic hyperglycemia on EDC-derived EVs. EVs are cell-derived vesicles enclosing proteins and nucleic acids that are naturally secreted as a means of cell-to-cell communication 14. Recently, EVs produced by cardiac-derived cells have been shown to prevent fibrosis while enhancing both angiogenesis and cardiomyocyte proliferation 15-17. We hypothesize that preventing the accumulation of reactive dicarbonyls through Glo1 overexpression will enhance EDC-mediated cardiac repair by rejuvenating the production of pro-healing EVs.

## Methods

### Cell culture

All animal protocols were reviewed and approved by the University of Ottawa Animal Care Committee. Male and female murine cardiac tissue was obtained from 3.4±1.3 month old wild-type C57Bl/6 (WT) or C57Bl/6-PEP8-Glo1 (Glo1) transgenic mice 18 under isoflurane sedation. EDCs were cultured as described previously 6, 19-24. Briefly, cardiac tissue was minced, enzymatically digested with collagenase (1mg/mL, ThermoFisher) and plated as cardiac explants on fibronectin coated dishes (ThermoFisher). Cardiac explants were cultured in physiological 5% oxygen conditions using custom formulated glucose-free Iscove's Modified Dulbecco's Medium supplemented with 5 mmol/l D-glucose (physiological glucose), 20 mmol/l D-mannitol, 20% fetal bovine serum, 10% penicillin streptomycin, 2 mmol/l L-glutamine, and 0.1 mmol/l 2-mercaptoethanol (all from ThermoFisher). EDCs that emigrated from the plated tissue were harvested using mild trypsinization (0.05% trypsin; ThermoFisher) once a week every 4 weeks for direct experimentation. Human umbilical vein endothelial cells (HUVECs, Lonza) were cultured in standard media (CC-2517, Lonza) at 21% oxygen conditions according to the manufacturer's directions. Neonatal rat ventricular myocytes (NRVMs) were prepared as described from 2 day old Sprague-Dawley rats (Harlan) 25, 26.

### Streptozotocin induced hyperglycemia

Hyperglycemia was induced by intraperitoneal injection of streptozotocin (STZ, 50 mg/kg for 5 days, Millipore Sigma) in 0.05 M sodium citrate (Fisher Scientific). Non-hyperglycemic control mice received equal volumes of 0.05 M sodium citrate. Fasting blood glucose and glycated hemoglobin (HbA1c) measurements were measured prior to sacrifice for enzyme- linked immunosorbent assay (CSB-E08141m, Cusabio).

### Cell metabolism

Bioenergetic determinations were performed using Agilent-Seahorse XF24 analyzer 27. One day prior to experiment, the assay cartridge was hydrated overnight using XF calibrant solution. The next day, cells were placed in Seahorse assay medium (bicarbonate-free Dulbecco's Modified Eagle Medium, 5mM D-glucose, 4 mM L-glutamine, 1mM sodium pyruvate; Millipore Sigma) and incubated in a non-carbon dioxide incubator at 37 degrees Celsius for 30 minutes. Oxygen consumption rate (OCR) and extracellular acidification rate (ECAR) measurements were determined at basal levels and following injection of 2 μM oligomycin, 1 μM carbonyl cyanide-4 (trifluoromethoxy) phenylhydrazone and 1 uM antimycin A (Millipore Sigma). These injections allow the measurements of leak respiration, maximal respiration and non-mitochondrial respiration respectively. At the end of the run, EDCs were lysed with 50μl of 0.5 M NaOH for protein quantification (Millipore Sigma). Rates were normalized to protein content in each well.

### Measurement of proliferation, reactive oxygen species content, anti-oxidant reserves, senescence and apoptosis resistance

Cell growth within 1% oxygen 1% serum 5% (physiological) glucose conditions was quantified using a colorimetric assay (Dojindo) with confirmatory manual haemocytometer cell counts. Hydrogen peroxide is a major form of reactive oxygen species (ROS) and its level was assayed in cell at 5% oxygen with 20% serum physiological glucose conditions using 2',7-dichlorofluorescein diacetate fluorescent emission (ab113851, Abcam). Cell senescence within 5% oxygen 1% serum physiological glucose conditions was quantified using senescence-associated β-galactosidase activity (KAA002, EMD Millipore). The percentage of β-galactosidase cells was quantified in five random fields per cell line assayed. The number of apoptotic cells within 1% oxygen 1% serum physiological glucose conditions cultured with 0.1µM staurosporine (Millipore Sigma) was quantified using flow cytometry (Guava easyCyte, Luminex) for phycoerythrin-Annexin V (PE-Annexin V) and 7-Aminoactinomycin D (7-AAD; 559763, BD Biosciences).

### *In vivo* cardiac repair

The ability of transplanted EDCs to promote cardiac function after permanent left coronary artery (LCA) ligation was evaluated using male murine EDCs injected into female wild-type C57 mice 6, 19-24. Animals were injected with buprenorphine (0.05 mg/kg; subcutaneous) 1 hour prior to surgery and twice daily thereafter for 3 days. During the surgery, mice were intubated, anesthetized using isoflurane (maintained at 2-3%) and maintained under physiological temperatures. One week after LCA ligation, animals were randomized to receive 1x10^5^ EDCs or vehicle injected into the infarct border zone using echocardiographic guidance 6, 19-24. Myocardial function was evaluated using echocardiography (VisualSonics V1.3.8) and invasive hemodynamics (Transonic ADV500). During intramyocardial injection of cells/vehicle or physiological measures, animals were intubated, anesthetized using isoflurane (maintained at 2-3%) and maintained under physiological temperatures. After the final echocardiogram, the hearts were excised and randomly allocated to histological analysis or quantitative polymerase chain reaction for retained male (transplanted) cells 28. Hearts allocated to histology were fixed with 4% paraformaldehyde, embedded and sectioned. Tissue viability within the infarct zone was calculated from Masson's trichrome stained sections by tracing the infarct borders manually and then using ImageJ software to calculate the percent of viable myocardium within the overall infarcted area. To evaluate proliferation and differentiation, sections were co-stained with bromodeoxyuridine (BrdU; 11778-1-AP, Proteintech) and cardiac troponin T (cTnT; ab66133, Abcam). Capillary density within the infarct border zone was assessed using isolectin B4 staining (B-1205; Vector Laboratories) in conjunction with 4′,6-diamidino-2- phenylindole (Millipore Sigma). The total number of nuclei within one image field of the border zone were counted and assessed for marker expression 6, 19-24.

### Effects of conditioned media on angiogenesis and cardiomyogenesis

Conditioned medium was prepared from confluent cultures after 48 hours within 1% oxygen, and 1% serum under physiological glucose conditions in the presence and absence of 20 μM GW4869 (Millipore Sigma). The angiogenic potential of EDC conditioned media was evaluated using a cytokine depleted matrigel assay (ECM625, Millipore Sigma) 5, 6, 21, 22. All phase contrast fields were compiled, and cumulative tubular growth was determined using Image J (National Institutes of Health). To evaluate the effect of EDC conditioned media on myocyte survival/proliferation, NRVMs were cultured in 10 μM BrdU (Thermo Fischer Scientific) prior to flow cytometry for BrdU incorporation (ab6326, Abcam).

### Extracellular vesicle collection, analysis and RNASeq profiling

Conditioned medium was collected after 48 hours of culture within 5% oxygen and 1% exosome- free serum under physiological glucose conditions. EVs were isolated using ExoQuick-TC exosome precipitation solution (System Biosciences) for EV tracking analysis (NanoSight LM10; Malvern Instruments). Micro and total RNA were extracted (Qiagen) and a small RNA library was prepared (Lexogen) for RNASeq evaluation of expression profiles (Illumina NextSeq500). Quality assessment was performed using FastQC (Babraham Institute) and reads were trimmed with a minimum sequence length of 18bp for mapping using bowtie (v1.1.2) aligned to the mus musculus mature microRNA (miRNA) sequences. Differential expression analysis was performed using the negative binomial model of read counts implemented in the DESeq2 R library. Principal component analysis was applied to the matrix of gene expression values (read counts) to identify the major components of gene expression variation. Hierarchical clustering was calculated using Euclidian distance between rlog-transformed normalized count values for all transcripts.

### Data and statistical analysis

All procedures and analyses were performed blinded to animal or cell identity. All data are presented as mean ± SEM. To determine if differences existed within groups, data was analyzed by a one-way or two-way ANOVA, as appropriate. If such differences existed, Sidak's multiple comparisons test was used to determine the group(s) with the difference(s) (Prism 8.00; GraphPad Software, Inc.). A final value of P≤0.05 was considered significant for all analyses.

## Results

### Glo1 reverses the adverse effects of hyperglycemia on cell function

**Figure 1:**
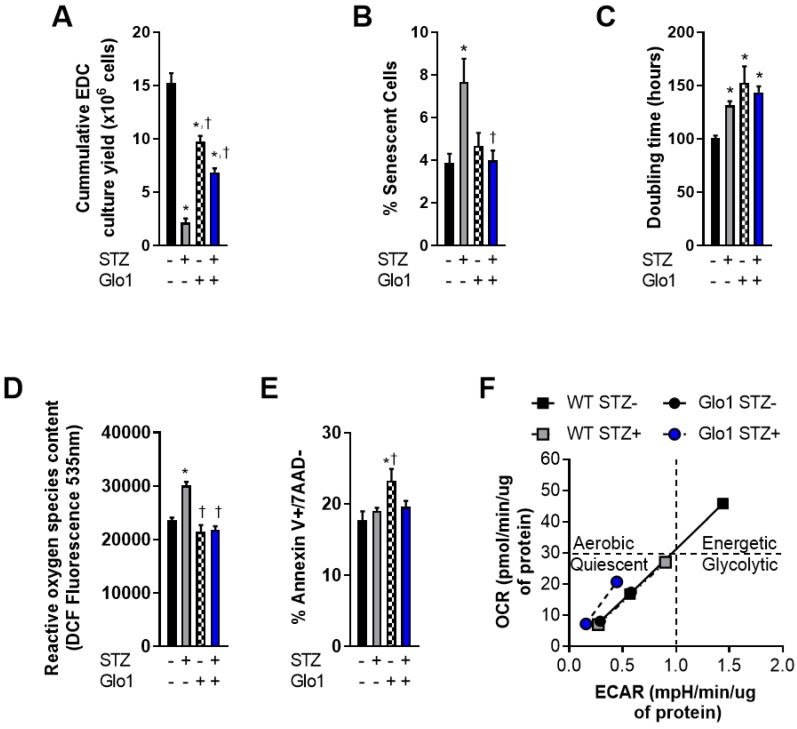
** Overexpression of Glo1 attenuates the effects of hyperglycemia on heart explant-derived cell function.** (**A**) Cumulative culture yield of EDCs from normoglycemic WT (n=10), STZ-treated WT (n=10), normoglycemic Glo1 (n=10) and STZ-treated Glo1 (n=10) murine hearts under physiological (20% serum, 5% oxygen) culture conditions. (**B**) Proportion of β-galactosidase+ (senescent) cells using random field imaging within cultures (n=12/group). (**C**) Population doubling times of normoglycemic WT (n=8), STZ-treated WT (n=8), normoglycemic Glo1 (n=8) and STZ-treated Glo1 (n=8) EDCs after 48 hours in 1% serum 1% oxygen culture conditions. (**D**) Basal reactive oxygen species quantification within EDCs cultured from hyperglycemic (n=6) and non-hyperglycemic (n=6) biopsies. Data is expressed as the dichlorofluorocein-diacetate (DCF) fluorescence intensity. (**E**) Apoptotic susceptibility of EDCs measured using flow cytometry for annexin V and 7-Aminoactinomycin D (7AAD) expression after 24 hours incubation with 0.1µM staurosporine (n=12). Data indicate mean ± SEM; *p<0.05 versus WT STZ- EDCs; †p<0.05 versus WT STZ+ EDCs. (**F**) Cellular metabolic phenotyping in normoglycemic and hyperglycemic EDCs. OCR: Oxygen Consumption Rate; ECAR: Extracellular Acidification Rate.

EDCs are the early cell product collected from plated cardiac biopsies prior to antigenic selection 29 or culture guided expansion 10. As such, they provide the ideal platform to investigate donor dependent effects before ex vivo culture artefacts. In this report, EDCs were cultured from the cardiac biopsies of 16-week old WT or Glo1 overexpressing mice 2 months after treatment with STZ or vehicle. Fasting blood glucose obtained at the time of sacrifice was markedly higher in STZ treated animals as compared to vehicle treated controls (22.2±1.0 versus 6.6±0.2 mmol/L, p≤0.001) and resulted in a 2.0±0.1 fold increase in glycated hemoglobin (p=0.001 versus vehicle treated mice) within both WT and Glo1 STZ treated mice. Despite having no influence on the amount of tissue available for plating, chronic hyperglycemia reduced the cumulative number of EDCs cultured from plated cardiac biopsies by 86±3% (p=0.0009 versus WT vehicle treated controls; Fig. 1A); an observation in part attributable to the ~2 fold greater number of senescent EDCs found in cell lines sourced from STZ-treated WT mice (p=0.03; Figs. 1B and S1). This effect was not limited solely to the initial proliferation of cells from plated tissue as hyperglycemia also prolonged the population doubling time of WT EDCs by 30±4% (p=0.04 versus normoglycemic WT EDCs; Fig. 1C). Consistent with previous work 5, hyperglycemia also increased the reactive oxygen species content within WT EDCs, as measured by dichlorofluorocein-diacetate (Fig. 1D) but did not alter the ability of these cells to withstand stress culture conditions (Fig. 1E).

Unexpectedly, cell culture yields from control normoglycemic tissue were decreased (36±8% fewer cells, Fig. 1A) while population doubling times were increased (1.5±0.2 fold greater, Fig. 1C) in Glo1 EDCs as compared to WT EDCs. Given that somatic gene transfer of Glo1 has no effect on these parameters 5, the possibility that these differences may be attributable to unforeseen inbred alterations in cell metabolism was probed using an analysis of mitochondrial respiration (oxygen consumption rate) in response to glycolytic activity (extracellular acidification rate).

As shown in Fig. 1F, WT normoglycemic EDCs have normal resting and maximal oxidative and glycolytic metabolic activities, consistent with the conclusion that there is a high degree of metabolic flexibility. Metabolic flexibility was lacking in hyperglycemic EDCs treated with streptozotocin. Even the baseline rates of oxidative and glycolytic activities were lower compared to WT normoglycemic EDCs (Fig 1F: grey square, bottom left). Consistent with metabolic impairments, normoglycemic Glo1 EDCs displayed lower baseline oxidative and glycolytic rates (each approx. 50% or WT baseline values) that were not drastically altered with streptozotocin treatment. Moreover, the metabolic challenge resulted in a minimal increase in oxidative and glycolytic activities, thus demonstrating impaired metabolic flexibility, when compared to WT EDCs. These data suggest that the Glo1 transgenic line is metabolically inferior to WT cells. It follows, that any adverse effect from hyperglycemia on cell function or survival should be magnified in these cells. Interestingly, hyperglycemia had no effect on the baseline rates or rates upon metabolic stress in Glo1 EDCs.

### Glo1 attenuates the adverse effects of hyperglycemia on EDC-mediated repair of injured myocardium

**Figure 2:**
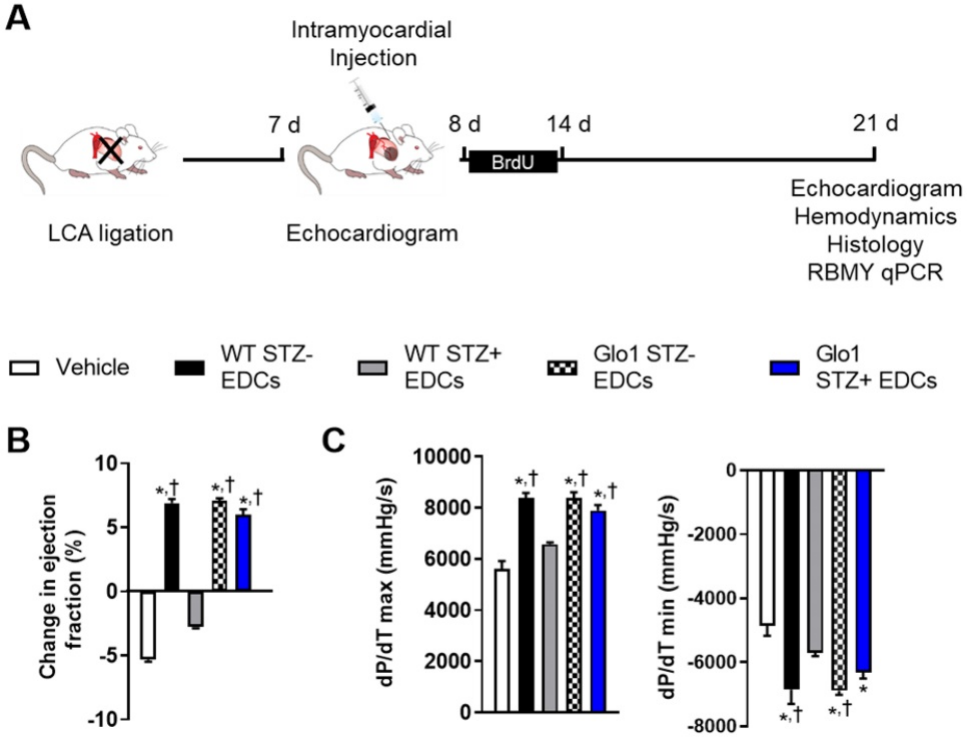
** Glo1 overexpression prevents the adverse effects of chronic hyperglycemia on heart explant-derived cell repair of ischemic myocardium. (A)** Experimental schemata. **(B)** Effect of cell treatment on echocardiographic left ventricular ejection fraction 4 weeks after left coronary artery (LCA) ligation. **(C)** Effects of cell treatment on invasive hemodynamic measures of contractility (left panel dP/dT max) and relaxation (right panel dP/dT min). Data indicate mean ± SEM; n=11; *p<0.05 versus vehicle; †p<0.05 versus WT STZ+ EDCs.

The influence of Glo1 overexpression on EDC-mediated repair of injured myocardium was explored using mice randomized to cell or vehicle injection 1 week after LCA ligation (Fig. 2A). As shown in Table S1, cardiac dimensions and function were similar at the time of randomization. Three weeks after receiving vehicle, cardiac function declined as adverse remodeling ensued (Fig. 2B and Table S1). Hyperglycemia impaired the ability of WT EDCs to promote myocardial repair as evidenced by markedly reduced echocardiographic (Fig. 2B and Table S1) and hemodynamic (Fig. 2C and Table S2) measures of myocardial function as compared to treatment with normoglycemic WT EDCs. Glo1 overexpression prevented chronic hyperglycemia from adversely influencing the ability of EDCs to promote myocardial function (p=ns compared to normoglycemic WT or normoglycemic Glo1 EDCs).

**Figure 3:**
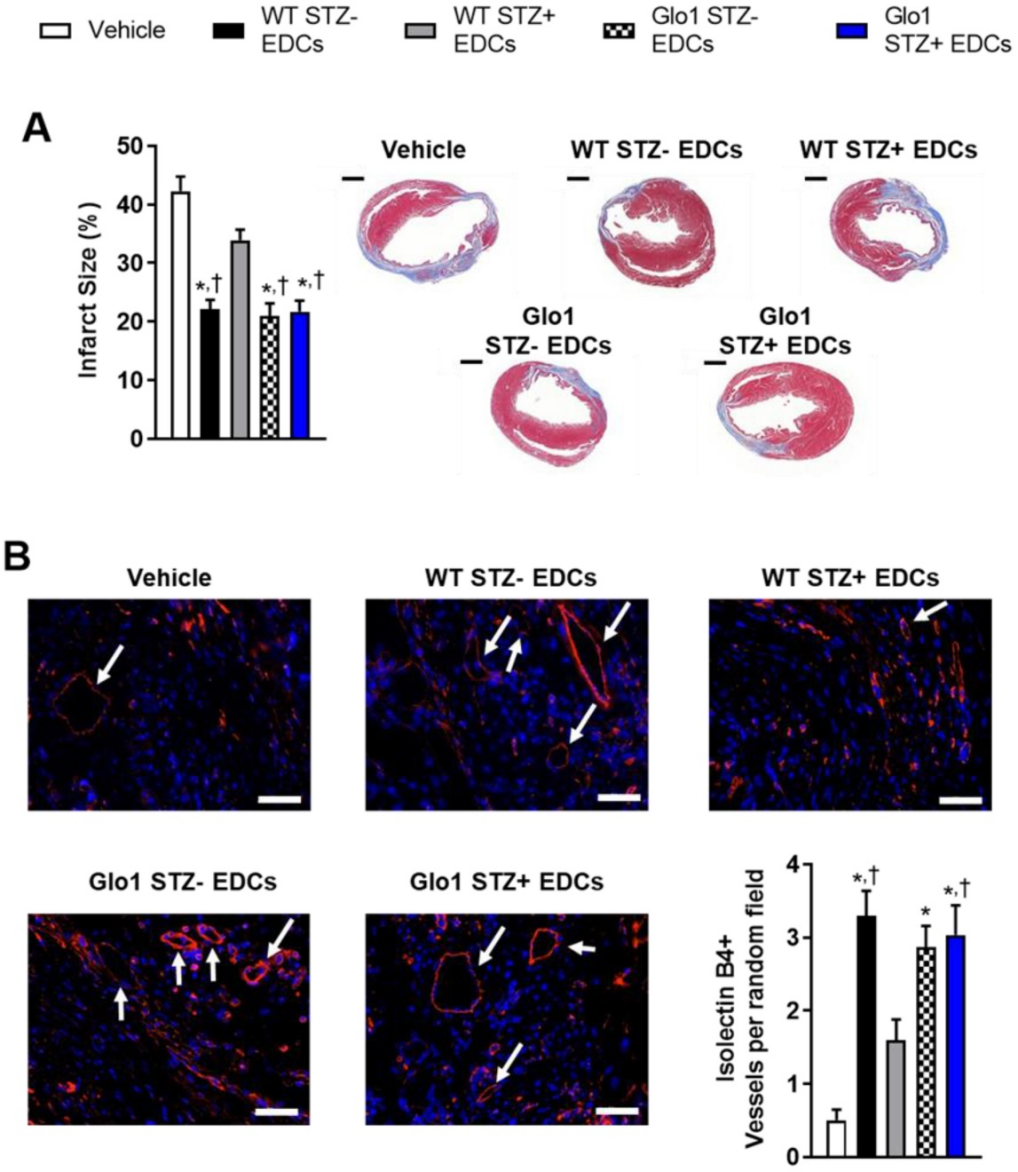
** Glo1 overexpression reduces the adverse effects of chronic hyperglycemia on cell-mediated ventricular remodeling and stimulation of endogenous repair by heart explant-derived cells. (A)** Effects of EDC treatment on infarct size 4 weeks after left coronary artery ligation (left panel) and representative images (right panel). Scale bar = 1000 µm. **(B)** Effects of EDC treatment on vessel density within treated hearts 4 weeks after LCA ligation. Representative isolectin B4+ images of each cell therapy 3 weeks after myocardial infarction. DAPI (blue). Isolectin B4 (red). Arrows indicate examples of vessels used for quantification. Scale bar = 50 µm.

**Figure 4:**
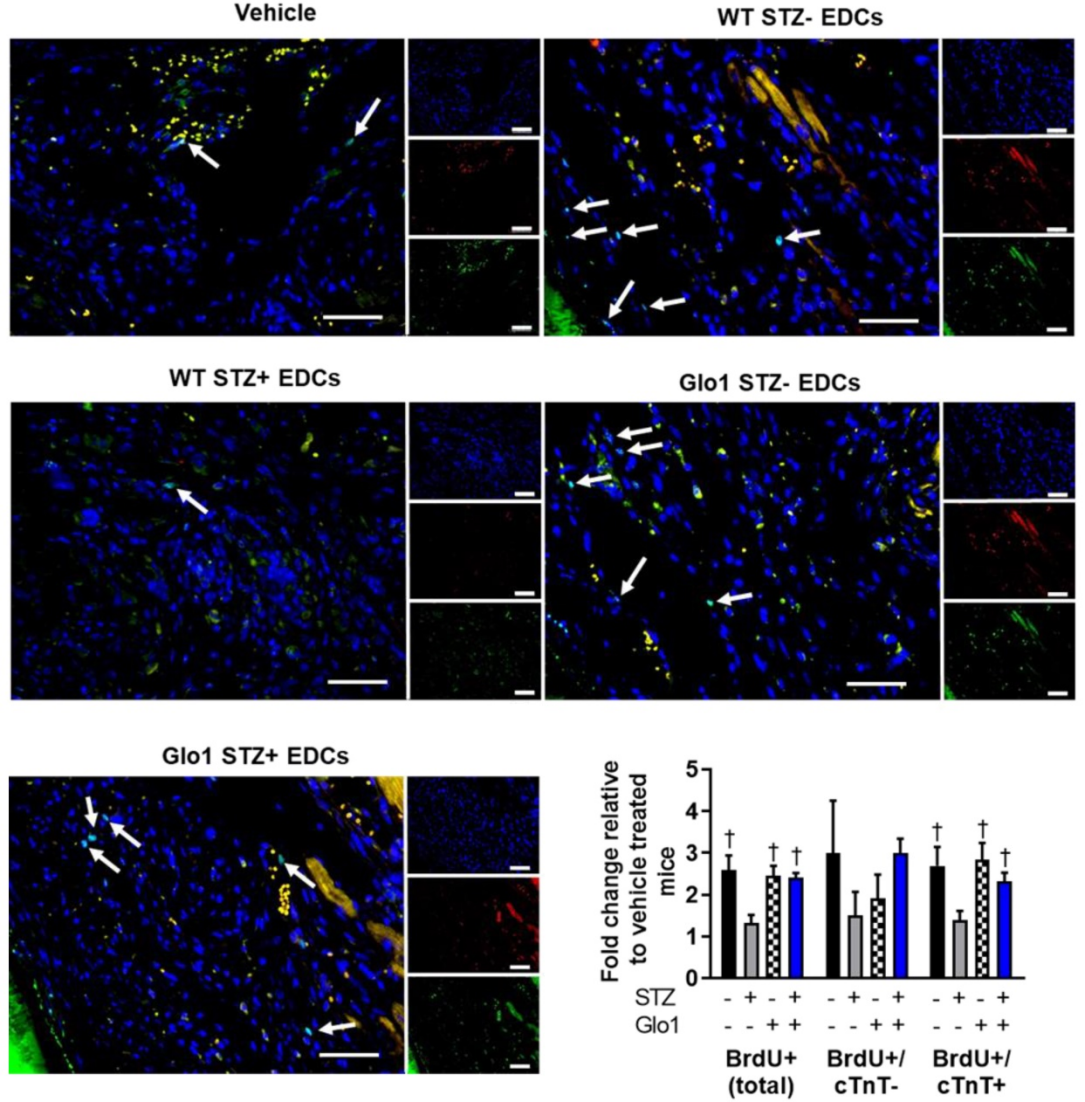
** Effect of Glo1 and hyperglycemia EDC generation of new cardiomyocytes.** Representative BrdU+ images of each cell therapy 3 weeks after myocardial infarction. DAPI (blue). Cardiac troponin T (red). BrdU (green). Scale bar = 50 µm. Random field analysis of new BrdU+/cTNT+ myocytes on histological sections 4 weeks after left coronary artery ligation ligation. Data indicate mean ± SEM; n=5-7; *p<0.05 versus vehicle; †p<0.05 versus WT STZ+ EDCs.

Hyperglycemia had similar effects on the ability of EDCs to prevent ventricular scarring as shown by the considerably larger ventricular scars found within histological sections from mice transplanted with hyperglycemic WT EDCs as compared to normoglycemic WT controls (Fig. 3A). Intra-myocardial injection of EDCs from mice that over-expressed Glo1 demonstrated infarct sizes equivalent to animals treated with normoglycemic WT EDCs. Immunohistochemistry revealed that animals that received EDCs from hyperglycemic WT donors had fewer vessels within the peri-infarct region (Fig. 3B). The effect of hyperglycemia on EDC-mediated vessel formation were abrogated when animals received EDCs from donors that over-expressed Glo1. As shown in Fig. 4, chronic hyperglycemia also decreased the ability of EDCs from hyperglycemic WT mice to promote the generation of new cardiomyocytes (BrdU+/cTnt+). Overexpression of Glo1 protected EDCs from the effects of hyperglycemia as EDCs stimulated proliferation of endogenous cells akin to normoglycemic WT EDCs. Interestingly, qPCR analysis of ventricular lysates failed to detect any retained transplanted cells 3 weeks after intra- myocardial injection suggesting the observed changes in cardiac function occurred in the absence of appreciable increases in long-term engraftment (data not shown). Thus, Glo1overexpression attenuates the adverse effects of hyperglycemia to enhance therapeutic regeneration through stimulation of angiogenesis and the generation of new cardiomyocytes.

### Glo1 limits the effects of hyperglycemia on EDC-mediated angiogenesis by enhancing extracellular vesicle production

**Figure 5:**
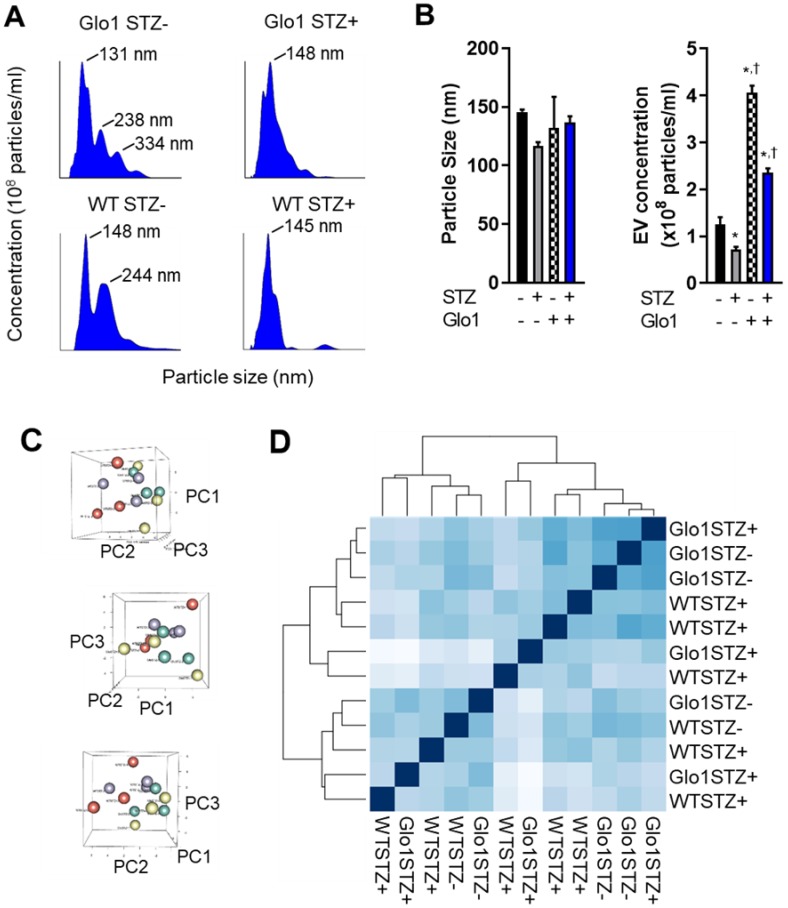
** Chronic hyperglycemia and Glo1 overexpression alter extracellular vesicle production by heart explant-derived cells. (A)** Representative Nanosight tracings of extracellular vesicles isolated from WT or Glo1 mouse EDCs 8 weeks after treatment with buffer or STZ with the size of the key peaks annotated. **(B)** Effects of chronic hyperglycemia and Glo1 overexpression on EV content (right panel) and size (left panel) within media conditioned within 1% (exosome-free) serum 1% oxygen conditions using NanoSight Tracking Analysis. Data indicate mean ± SEM; n=6, *p<0.05 versus non-STZ WT; †p<0.05 versus WT+STZ. **(C)** Three dimensional plots of the principle component analysis to the matrix of gene expression values demonstrating a lack of clear differences when samples are plotted on their first three principal components (PC). WT STZ- = purple symbols, WT STZ+ = red symbols, Glo1 STZ- = green symbols and Glo1 STZ+ = yellow symbols. **(D)** Hierarchical clustering calculated using Euclidian distance between rlog-transformed normalized count values for transcripts from EVs produced by WT STZ-, WT STZ+, Glo1 STZ- or Glo1 STZ+ EDCs.

Given the evanescent nature of transplanted cardiac-derived cells and the important role of indirect cardiac repair in cell treatment outcomes, the effects of hyperglycemia on the paracrine profile of EDCs was evaluated using media conditioned under low serum hypoxic conditions designed to mimic the harsh post- infarct environment. Unlike other medical comorbidities 6, 21, chronic hyperglycemia has negligible effects on cytokine production by EDCs 5. Therefore, we explore the impact of glycemic status on the production and contents of EVs which have been shown to mediate many of the actions of heart-derived cells 16. As shown in Figs. 5A and 5B, hyperglycemia markedly the decreased production of 133±7 nm EVs (43±6% fewer exosomes secreted, p=0.04 vs normoglycemic WT EDCs). Glo1 overexpression increased EV production by normoglycemic EDCs while attenuated the effects of chronic hyperglycemia on EV production. Plotting the first three principal components calculated from the RNASeq data of all 12 samples did not discriminate between samples; suggesting a lack of clear differences in expression between the conditions (Figure 5B). As shown in Figure 5C, hierarchical clustering of the Euclidian distance between rlog-transformed normalized count values for all transcripts recapitulated the principal component analysis and highlighted the observation that Glo1 expression and STZ treatment had no clear effect on the miRNA cargo within EDC EVs. Given this indistinct clustering, it was unsurprising that STZ treatment decreased only a single transcript within EVs produced by EDCs from Glo1 mice (MiR-146a, log2 fold change -3.2±0.8, p<0.05 versus WT EDC EVs). Although inspection miRNA expression based on unadjusted p-values implies that increasing count density might eventually uncover more differential expressed miRNAs (Table S3-S8), these would be of “low-fold change” and, with the severe reductions in EV production noted, are unlikely to account for a significant portion the observed differences in regenerative efficacy.

**Figure 6:**
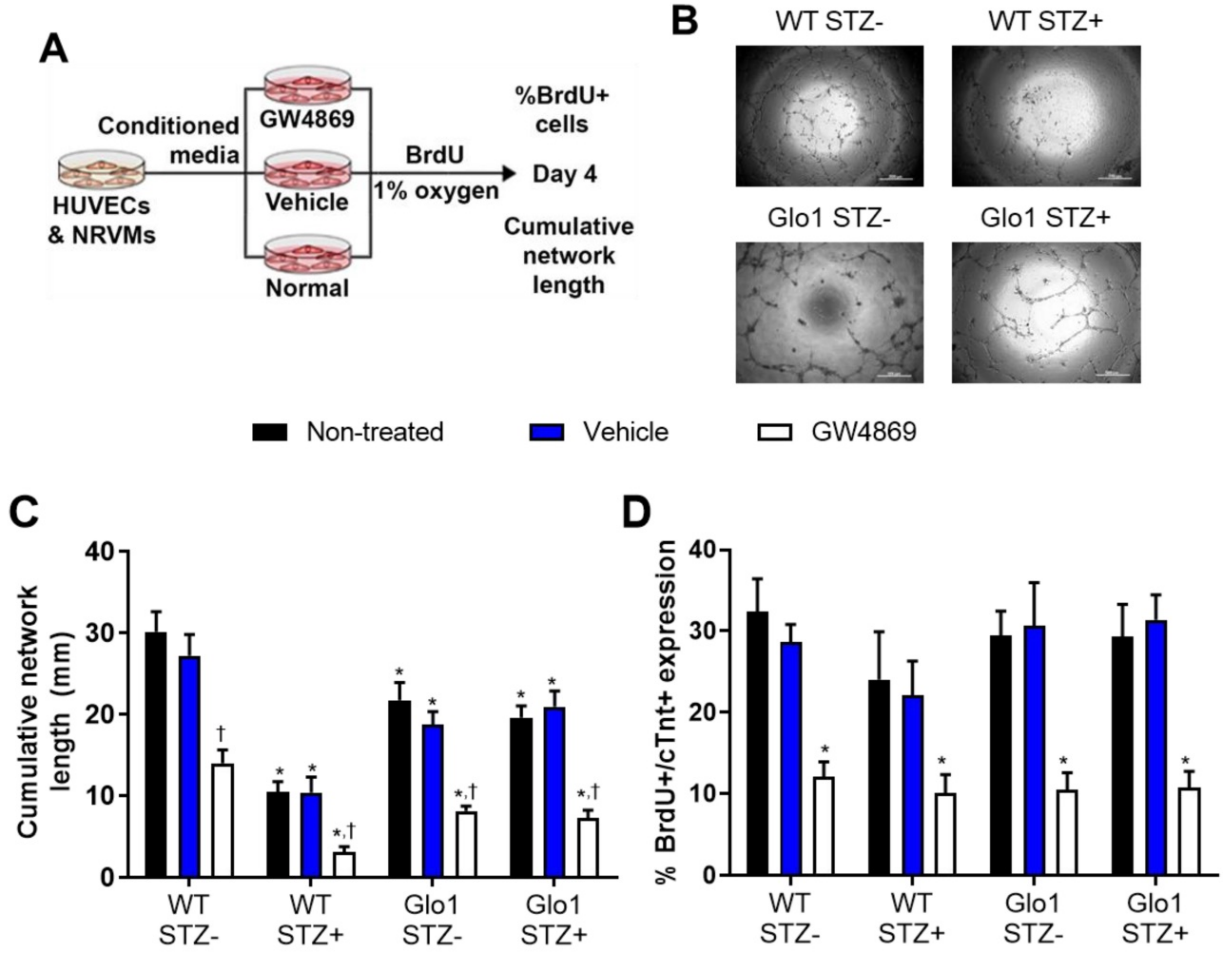
** Extracellular vesicles mediate the angiogenic and cardiomyogenic effects of EDCs. (A)** Schematic diagram outlining the application of media conditioned in the presence of EV release blocker GW4869, vehicle (dimethyl sulfoxide) or no treatment on the ability of HUVECs to form networks and NRVMs to generate new myocytes (BrdU+/cTNT+ cells).**(B)** Representative images of vascular network formation by HUVECs after 16 hours of exposure to EDC conditioned media. Scale bar = 500 µm. **(C)** Effects of EDC conditioned media on cumulative vascular network formation by HUVECs. Data indicate mean ± SEM; n=6, *p<0.05 versus non-STZ WT EDCs that received the same treatment during generation of conditioned media (non-treated, vehicle or GW4869); †p<0.05 versus EDCs from the same cohort (WT STZ-, WT STZ+, Glo1 STZ+ or Glo1 STZ-) that were not treated or were exposed to vehicle during generation of conditioned media. **(D)** Effects of EDC conditioned media on newly generated myocytes (BrdU+/cTNT+ cells) by NRVMs. Data indicate mean ± SEM; n=6, * p<0.05 versus EDCs from the same cohort (WT STZ-, WT STZ+, Glo1 STZ+ or Glo1 STZ-) that were not treated or were exposed to vehicle during generation of conditioned media.

The functional implications of hyperglycemia- induced changes in EV content was experimentally tested using established models of angiogenesis and cardiomyogenesis 5, 30, 31. To emulate the paracrine effects of EDCs on endothelial (HUVECs) and cardiomyocytes (NRVMs), EDC conditioned media was generated in hypoxic (1% oxygen) basal media conditions (Figure 6A). The contribution of EVs to the paracrine effect of EDCs was dissected by exposing cells to the neutral sphingomyelinase inhibitor GW4869 to block EV release during generation of conditioned media 16. Consistent with previous results 5, chronic hyperglycemia reduced the ability of EDC conditioned media to promote vascular network formation by 71±2% as compared to WT non-STZ EDC conditioned media (Figs. 6B and 6C). Although media conditioned by Glo1 mice EDCs demonstrated a reduced ability to stimulate tubule formation, Glo1 overexpression attenuated the anti-angiogenic effects of hyperglycemia. Consistent with the notion that EVs play a major role in the pro-angiogenic effects of EDCs, treatment with GW4829 reduced network formation in all cells treated regardless of glycemic status. In contrast to the angiogenic findings, hyperglycemia and Glo1 overexpression had no impact on the ability of EDC conditioned media to stimulate NRVM proliferation (BrdU+/cTNT+ cells; Fig. 6D). This effect was clearly linked to EV production as pre-treatment with GW4829 uniformly reduced BrdU+/cTNT+ cell content. Despite the many fold increase in EV production by Glo1 EDCs, neither angiogenesis nor cardiomyogenesis increased which suggests a threshold stimulatory capacity had been reached by the WT normoglycemic cells.

## Discussion

Over the past decade, heart-derived cell therapy has developed rapidly as a promising therapeutic for heart damage. Although early clinical trials focused on autologous (self to self) therapy and uniformly showed these products to be safe with promising hints of efficacy 1-4, the prospect of allogeneic (unrelated donor) therapy tempted investigators to explore allogenic approaches with mixed results 32, 33. Recently, an interim analysis of data from a large phase 2 trial demonstrated a low probability (futility) of achieving a statistically-significant difference in the primary efficacy endpoint 34. This discovery has renewed interest in exploring the therapeutic potential of autologous cell sources as, perhaps tellingly, autologous transplants (in the forms of bone marrow transplant) are the most common form of cell transplantation worldwide. Akin to other adult cell products, donor comorbidities have adverse influences on the regenerative efficacy of heart-derived cells and motivates study of these modifiers 6, 21. Previously, we have shown that chronic hyperglycemia promotes the generation of toxic methylglyoxal and attenuates the pro-angiogenic potential of EDCs 5. In this study, we explore the influence of the key methylglyoxal detoxification enzyme, Glo1, on chronic hyperglycemia induced EDC dysfunction.

We found that chronic hyperglycemia decreases both EDC culture yields and the ability of these cells to withstand oxidative stress or senescence. Intramyocardial injection after cardiac damage also demonstrated that hyperglycemia reduces the angiogenic, mitotic and anti-scarring properties of EDCs. Unlike other comorbidities (such as advanced age or hypertension) 6, 21, chronic hyperglycemia does not influence the cytokine signature of EDCs but markedly impaired the ability of EDCs to secrete pro-healing EVs. Importantly, this is the first study to identify the fundamental mechanism underlying reduced functional repair seen after transplant of cardiac 5 and non-cardiac cells 13, 35-37 from hyperglycemic donors.

Several years ago, we demonstrated that “high glucose” culture conditions (25 mM) significantly impacted the pro- angiogenic capacity of non-diabetic EDCs 5. Lowering the glucose content within media to a more physiologic range (5 mM) increased cell-mediated improvements in cardiac function, reduced scar sizes and infarct vascularization. Importantly, the “high glucose” conditions used represents the industry standard for mammalian cell culture which originated from reports over 30 years ago documenting that non-physiological conditions enhanced hematopoietic cell proliferation 38. To eliminate the possibility that non-physiologic media may be toxic to diabetic cells, this study performed all cell culture using custom media formulations conditions and is the first study to show the adverse effects of chronic hyperglycemia persist despite physiologic culture conditions (5 mM glucose and 5% oxygen) 39, 40.

From previous work, we suspected that methylglyoxal detoxification might attenuate the adverse effects of chronic hyperglycemia 5. Methylglyoxal is a toxic by-product formed at a relatively high flux through degradation of glyceraldehyde-3- phosphate and dihydroxyacetone phosphate. Increased oxidative stress is linked to dicarbonyl stress and advanced glycation end product (AGE) formation and glycated proteins to result in mitochondrial dysfunction, cell apoptosis, increased ROS/oxidative damage, and upregulation of the receptor for advanced glycation end products (RAGE) 41-43. Given that 99% of methylglyoxal is metabolized by Glo1, this represented an attractive target to study the impact of methylglyoxal burden on cell metabolism and performance. Prior work using transgenic strains has shown Glo1 overexpression attenuates hyperglycemia-induced endothelial dysfunction, oxidative damage and renal damage 44-46. Pertinent to this study, Glo1 overexpression restores the pro-angiogenic capacity of bone marrow-derived circulating angiogenic cells from hyperglycemic donors to promote ischemic hindlimb salvage 47. Consistent with our study findings, previous work has shown transgenic Glo1 overexpression reduces production of methylglyoxal-H1 (a principal methylglyoxal AGE) after myocardial infarction 46, increases peri-infarct neovascularization and prevents the development of diabetic cardiomyopathy 48.

In the course of the study, we unexpectedly found that our inbred Glo1 mice displayed impaired cellular metabolic characteristics which may be related to secondary effects of high levels of Glo1 overexpression such as altered glutathione and/or NADH redox potentials, or the shunting of glycolytic 3-carbon metabolites into the methylglyoxal pathway, away from oxidative pathways. Despite this tendency, Glo1 overexpression markedly attenuated the impact of hyperglycemia on post infarct repair by increasing EV production and endogenous repair which suggests these adverse metabolic derangements were more than compensated by the cytoprotective effects conferred by prevention of methylglyoxal overload. As such, we provide the first proof that reducing methylglyoxal content during chronic hyperglycemia prevents the adverse effects of hyperglycemia.

We also noted a marked disparity in the EV nanoparticle content within media conditioned by normoglycemic Glo1 or WT mice (almost 2-fold greater). Despite this, equivalent degrees of post infarct function and scar burden were seen. This observation supports our hypothesis that cardiac cell transplant outcomes are dependent on exposure to a potent paracrine cell product and, once a stimulation threshold is achieved, simply increasing the number of cells retained or EVs provided has no further benefits 19, 49. It follows that interventions broadening the cytokine 21-24 or EV 19 signature of transplanted cells are needed to boost stimulation of endogenous repair mechanisms. Conversely, the adverse effects of chronic hyperglycemia could be overcome by doubling the number of cells administered but, given effects on proliferation and the expense/logistics of prolonged cell culture, the practicality of this strategy is debatable.

In terms of study limitations, the STZ model chosen reflects insulin deficient hyperglycemia while the majority of patients potentially in need of cell therapy are type 2 diabetics with both hyperglycemia and insulin resistance. Although the STZ model provides an unbiased platform to observe the effects of chronic hyperglycemia, further work to extend this data to patients with type 2 diabetes and ischemic cardiomyopathy are needed. Our *in vivo* study design employed a non-diabetic recipient to avoid confounding effects from recipient hyperglycemia but the regenerative performance of cells within the hyperglycemic host deserves further study as cell treatment outcomes are clearly leveraged on endogenous repair mechanisms. Finally, panning through ventricular lysate failed to demonstrate meaningful persistence of transplanted cells. Previous work has shown that long-term persistence of transplanted cells is very modest (1-2% of the initial injectate) 19, 20, 22, 23, 50. It is possible the number of cells remaining was below the detection threshold for the male-specific target used 28 but this result needs to be verified and future work to define the critical retention window leading to cell treatment effects needs to be established.

## Abbreviations

7AAD: 7-Aminoactinomycin D
AGE: advanced glycation end product
BrdU: bromodeoxyuridine
cTNT: cardiac troponin T
EDC: explant-derived cell
ECAR: extracellular acidification rate
EV: extracellular vesicles
Glo1: glyoxalase 1
HUVEC: human umbilical vein endothelial cells
LCA: left coronary artery
NRVM: neonatal rat ventricular myocytes
OCR: oxygen consumption rate
PE: phycoerythrin
RAGE: receptor for advanced glycation end products
STZ: streptozotocin
WT: wild-type
