# Supplementary material for: Glyoxalase 1 Prevents Chronic Hyperglycemia Induced Heart-Explant Derived Cell Dysfunction
Source: Theranostics. 2019 Aug 9;9(19):5720–30. doi: 10.7150/thno.36639 (PMC6735395; doi:10.7150/thno.36639)
Supplement: Supplementary file 1 — Supplementary figures and tables. [file thnov09p5720s1.pdf]

	Vehicle		WT STZ-		WT STZ+		Glo STZ-		Glo STZ+	
Weeks post LCA ligation	1	4	1	4	1	4	1	4	1	4
EDV (µl)	64±5	56±5	50±4	57±4	59±4	69±4	58±3	68±4	58±4	67±5
ESV (µl)	43±5	36±3	33±3	34±3	40±4	49±3	42±3	43±3	43±3	46±4
SV (µl)	21±1	19±1	17±2	23±2	19±1	20±1	17±1	24±1*	16±1	21±1
FAC (%)	21±2	18±2	21±1	25±1*	19±1	18±1	18±1	22±1	16±1	20±1
CO (ml/min)	8±1	7±1	7±1	9±1*	7±1	8±1	7±1	9±1*	7±1	9 ±1*

Table S1. Echocardiographic measurements of left ventricular function over the 4-week follow-up period. EDV= end diastolic volume, ESV = end systolic volume, SV = stroke volume, FAC = fractional area shortening, CO = cardiac output. Data are mean ± SEM; n=10-11; *p<0.05 vs. vehicle 4 weeks after LCA ligation.

	SW (mmHg*uL)	Vmax (uL)	Vmin (uL)	Ves (uL)	Ved (uL)	Pmax (mmHg)	Pmin (mmHg	Pmean (mmHg)
Vehicle	1237±107	43±4	21±4	25±4	38±4	83±2	5±1	34±1
WT STZ-	2108±230*	49±3	21±2	24±2	45±3	93±5*	1±1*	38±2
WT STZ+	1961±206*	54±5	22±4	25±4	50±5	81±1	4±1	30±2
Glo1 STZ-	2177±770*	55±20	26±9	28±10	52±18	91±32	2±1*	36±3
Glo1 STZ+	1646±182	48±4	23±3	26±3	43±3	86±1	1±1*	34±2

	Ped (mmHg)	Pdev (mmHg)	Pes (mmHg)	HR (bpm)	CE	Tau (ms)
Vehicle	9±1	78±2	76±3	501±13	0.6±0.1	8±1
WT STZ-	7±1	89±4*	89±5*	548±10	0.8±0.1	6±1*
WT STZ+	4±1	81±1	77±1	490±13	0.8±0.3	6±1*
Glo1 STZ-	5±2	89±3*	87±3*	519±183	0.7±0.3	6±1*
Glo1 STZ+	4±1*	85±1*	83±1	538±16	0.7±0.1	6±1*

	dV/dt max (uL/s)	dV/dt min (uL/s)	P@dP/dt max (mmHg)	V@dP/dt max (uL)	V@dP/dt min (uL)
Vehicle	952±87	-1034±80	45±2	40±4	23±4
WT STZ-	1175±133	-1570±245	52±2*	46±3	21±2
WT STZ+	1154±170	-1520±277	43±1	49±5	23±3
Glo1 STZ-	1022±361	-1411±499	50±18	53±9	27±9
Glo1 STZ+	1074±123	-1226±163	47±1	45±4	25±3

	PVA (mmHg*uL)	PE (mmHg*uL)
Vehicle	1800±179	562±104
WT STZ-	2707±190	599±55
WT STZ+	2562±311	627±139
Glo1 STZ-	3191±1128*	1014±358
Glo1 STZ+	2377±299	730±167

Table S2. Hemodynamic measurements of left ventricular function 4 weeks after LCA ligation. SW=stroke work, CO=cardiac output, SV=stroke volume, Vmax=maximum volume, Vmin=minimum volume, Ves=end systolic volume, Ved=end diastolic volume, Pmax=maximum pressure, Pmin=minimum pressure, Pmean=mean pressure, Pes=end systolic pressure, Ped=end diastolic pressure, HR=heart rate, Ea=arterial elastance, dV/dtmax=maximum derivative of volume, dV/dtmin=minimum derivative of volume, P@dP/dtmax=pressure at maximum derivative of pressure, V@dP/dtmax=volume at maximum derivative of pressure,

V@dP/dtmin=volume at minimum derivative of pressure, PVA=pressure-volume area, PE=potential energy, CE=cardiac events. Data are mean ± SEM; n=10-11; *p<0.05 vs. vehicle 4 weeks after LCA ligation.

miRNA	log2FoldChange	lfcSE	stat	P value	P adj
miR-210	2.312809	0.901019	2.566882	0.010262	0.947884
miR-34c	-2.50578	1.185529	-2.11364	0.034546	0.947884
miR-140	1.962702	0.962329	2.039532	0.041397	0.947884
miR-301a	1.362053	0.696508	1.955545	0.050519	0.947884
miR-341	1.71403	0.887146	1.932073	0.053351	0.947884
miR-154	3.512977	1.89919	1.849724	0.064353	0.947884
miR-329	2.477037	1.343973	1.843071	0.065319	0.947884
miR-381	1.699713	0.922642	1.842224	0.065442	0.947884
miR-155	-1.19513	0.651159	-1.83539	0.066448	0.947884
miR-1843a	-2.98193	1.653822	-1.80306	0.07138	0.947884
miR-129	-1.27829	0.720592	-1.77394	0.076073	0.947884
miR-369	2.146223	1.366976	1.570052	0.116403	0.947884
miR-212	-2.45111	1.566797	-1.56441	0.117722	0.947884
miR-101a	1.018416	0.657785	1.54825	0.121562	0.947884
miR-344b	-2.54504	1.659918	-1.53323	0.125219	0.947884
miR-21a	0.814414	0.534622	1.523344	0.127673	0.947884
miR-136	1.95559	1.314809	1.487357	0.136921	0.947884
miR-31	-0.71427	0.480299	-1.48714	0.136977	0.947884
miR-34a	1.146641	0.773557	1.482296	0.138262	0.947884
miR-487b	1.714169	1.169758	1.465405	0.142811	0.947884

Table S3. Effect of chronic hyperglycemia (STZ treatment) on the top 20 miRNAs expressed within EVs produced by Glo1 mice EDCs (i.e., Glo1TG STZ+ vs. Glo1TG STZ- mice). lfcSE = log fold change standard error.

miRNA	log2FoldChange	lfcSE	stat	pvalue	padj
miR-30b	-1.57454	0.543269	-2.89826	0.003752	0.382047
miR-344b	-4.66204	1.611704	-2.89262	0.00382	0.382047
miR-206	3.895296	1.485197	2.622748	0.008722	0.45393
miR-101a	1.780698	0.694085	2.565531	0.010302	0.45393
miR-19b	2.345239	0.939573	2.496069	0.012558	0.45393
miR-19a	2.671103	1.089332	2.452055	0.014204	0.45393
miR-34a	1.873565	0.785231	2.386006	0.017032	0.45393
miR-155	-1.48265	0.653239	-2.26968	0.023227	0.45393
miR-23a	-1.43331	0.631678	-2.26906	0.023265	0.45393
miR-93	1.298564	0.575955	2.254627	0.024157	0.45393
miR-434	-1.14165	0.522042	-2.18689	0.02875	0.45393
miR-26a	-0.95483	0.436776	-2.18609	0.028809	0.45393
miR-152	-1.10465	0.512119	-2.15702	0.031004	0.45393
miR-673	-2.09499	0.975671	-2.14723	0.031775	0.45393
miR-181c	-1.54121	0.734534	-2.09822	0.035886	0.478476
miR-146a	-1.64279	0.817675	-2.0091	0.044527	0.534092
miR-210	1.746985	0.901657	1.937528	0.052681	0.534092
miR-151	-1.11304	0.577436	-1.92756	0.05391	0.534092
miR-24	-1.45765	0.758215	-1.92247	0.054546	0.534092
miR-221	1.094898	0.570943	1.917701	0.055149	0.534092

Table S4. Effect of Glo1 overexpression on the top 20 miRNAs expressed within EVs produced by EDCs sourced from hyperglycemic mice (i.e., Glo1 STZ+ vs. WT STZ+ mice). lfcSE = log fold change standard error.

miRNA	log2FoldChange	lfcSE	stat	pvalue	padj
miR-146a	-3.22311	0.813221	-3.96339	7.39E-05	0.014779
miR-210	2.666109	0.904595	2.947296	0.003206	0.320566
miR-301a	1.920922	0.719257	2.670702	0.007569	0.386712
miR-146b	-2.22196	0.834239	-2.66346	0.007734	0.386712
miR-222	-1.82234	0.709968	-2.56679	0.010264	0.410574
miR-101a	1.653745	0.700512	2.360765	0.018237	0.549395
miR-155	-1.48915	0.65296	-2.28061	0.022572	0.549395
miR-34a	1.777719	0.784062	2.267321	0.023371	0.549395
miR-19a	2.430569	1.094283	2.221153	0.026341	0.549395
miR-19b	2.063495	0.93592	2.204777	0.02747	0.549395
miR-126a	-2.25143	1.053058	-2.13799	0.032517	0.591222
miR-140	1.950176	0.98604	1.977786	0.047953	0.754389
miR-206	2.904862	1.475834	1.968285	0.049035	0.754389
miR-34b	2.654031	1.403193	1.891422	0.058568	0.790817
miR-148b	-2.02541	1.074467	-1.88504	0.059424	0.790817
miR-29a	1.456742	0.784324	1.857322	0.063265	0.790817
miR-322	2.766194	1.512662	1.828693	0.067446	0.793477
miR-181a	1.390067	0.782257	1.776996	0.075569	0.839655
miR-673	-1.69412	0.994736	-1.70309	0.088552	0.915032
miR-3102	2.948882	1.774899	1.661436	0.096626	0.915032

Table S5. Effect of chronic hyperglycemia and Glo1 overexpression on the top 20 miRNAs expressed within EVs produced by EDCs (i.e., Glo1 STZ+ vs. WT STZ- mice). lfcSE = log fold change standard error.

miRNA	log2FoldChange	lfcSE	stat	pvalue	padj
miR-365	-3.5198	1.250558	-2.81458	0.004884	0.382832
miR-411	-1.49141	0.567827	-2.62653	0.008626	0.382832
miR-31	1.271455	0.487355	2.60889	0.009084	0.382832
miR-181c	-1.90796	0.733143	-2.60243	0.009256	0.382832
miR-154	-4.84412	1.869621	-2.59096	0.009571	0.382832
miR-152	-1.22033	0.50878	-2.39855	0.01646	0.485734
miR-329	-3.13197	1.335685	-2.34484	0.019035	0.485734
miR-434	-1.21422	0.521117	-2.33003	0.019805	0.485734
miR-30b	-1.22283	0.533327	-2.29283	0.021858	0.485734
miR-381	-2.05071	0.920522	-2.22777	0.025896	0.515644
miR-136	-2.82514	1.288686	-2.19226	0.02836	0.515644
miR-1249	-2.80849	1.379075	-2.0365	0.0417	0.688748
miR-23a	-1.23661	0.622802	-1.98555	0.047083	0.688748
miR-434	-1.53321	0.776114	-1.97549	0.048212	0.688748
miR-93	1.08521	0.575335	1.886221	0.059265	0.767101
miR-145a	-0.951	0.510438	-1.8631	0.062448	0.767101
miR-541	-0.76078	0.41688	-1.82494	0.068011	0.767101
miR-22	-0.827	0.454853	-1.81816	0.069039	0.767101
miR-344b	-2.117	1.209797	-1.74988	0.080138	0.772315
let-7e	0.94845	0.546765	1.734658	0.082801	0.772315

Table S6. Effect of chronic hyperglycemia or Glo1 overexpression on the top 20 miRNAs expressed within EVs produced by EDCs (i.e., Glo1 STZ- vs. WT STZ+ mice). lfcSE = log fold change standard error.

miRNA	log2FoldChange	lfcSE	stat	pvalue	padj
miR-146a	-2.49052	0.805069	-3.09355	0.001978	0.395549
miR-129	2.508394	0.939439	2.670099	0.007583	0.589971
miR-129	1.906925	0.728442	2.617812	0.00885	0.589971
miR-1843a	4.379817	1.743261	2.512428	0.01199	0.599519
miR-34c	3.025677	1.370071	2.208409	0.027216	0.983238
miR-673	3.3666	1.598156	2.106553	0.035156	0.983238
miR-222	-1.43067	0.685342	-2.08752	0.036841	0.983238
miR-130a	1.100039	0.561415	1.959403	0.050066	0.983238
miR-126a	-1.93741	0.998142	-1.94102	0.052256	0.983238
miR-374b	-3.42074	1.939228	-1.76397	0.077737	0.983238
miR-1249	-2.42113	1.393108	-1.73793	0.082223	0.983238
miR-151	-1.09547	0.637426	-1.71858	0.085691	0.983238
miR-501	-2.23875	1.304574	-1.71608	0.086148	0.983238
miR-148b	-1.7691	1.031686	-1.71477	0.086387	0.983238
miR-409	-2.61461	1.54876	-1.6882	0.091373	0.983238
miR-34b	-0.92064	0.620267	-1.48427	0.137737	0.983238
miR-136	-1.97556	1.33508	-1.47973	0.138945	0.983238
miR-100	-1.01635	0.697481	-1.45717	0.145069	0.983238
miR-1a	2.222876	1.547429	1.436496	0.150861	0.983238
miR-30c-2	1.313372	0.917658	1.431222	0.152367	0.983238

Table S7. Effect of Glo1 overexpression on the top 20 miRNAs expressed within EVs produced by EDCs (i.e., Glo1 STZ- vs. WT STZ- mice). lfcSE = log fold change standard error.

miRNA	log2FoldChange	lfcSE	stat	pvalue	padj
miR-222	-1.67882	0.709268	-2.36697	0.017934	0.968498
miR-193b	3.940389	1.704884	2.311236	0.02082	0.968498
miR-126a	-2.37131	1.059296	-2.23857	0.025184	0.968498
miR-100	-1.43443	0.699919	-2.04941	0.040422	0.968498
miR-21a	2.627277	1.301576	2.018535	0.043536	0.968498
miR-30b	1.078709	0.541293	1.992839	0.046279	0.968498
miR-31	-0.97366	0.491433	-1.98127	0.047561	0.968498
miR-3102	3.49785	1.765481	1.981245	0.047564	0.968498
miR-146a	-1.58032	0.804634	-1.96403	0.049527	0.968498
miR-1198	1.512205	0.777078	1.946014	0.051653	0.968498
miR-23a	1.225475	0.634936	1.930077	0.053597	0.968498
miR-146b	-1.50324	0.827949	-1.81562	0.069429	0.968498
miR-154	3.183718	1.778737	1.789875	0.073474	0.968498
miR-365	2.131486	1.204507	1.769592	0.076795	0.968498
miR-421	-2.07261	1.185083	-1.74891	0.080306	0.968498
miR-148b	-1.86204	1.065656	-1.74732	0.080582	0.968498
miR-431	1.693858	0.976296	1.734985	0.082744	0.968498
miR-126a	-2.4731	1.467592	-1.68514	0.091961	0.968498
miR-434	0.862126	0.522365	1.650428	0.098855	0.968498
miR-129	1.589731	0.96932	1.640047	0.100995	0.968498

Table S8. Effect of chronic hyperglycemia on the top 20 miRNAs expressed within EVs produced by EDCs (i.e., WT STZ+ vs. WT STZ- mice). lfcSE = log fold change standard error.

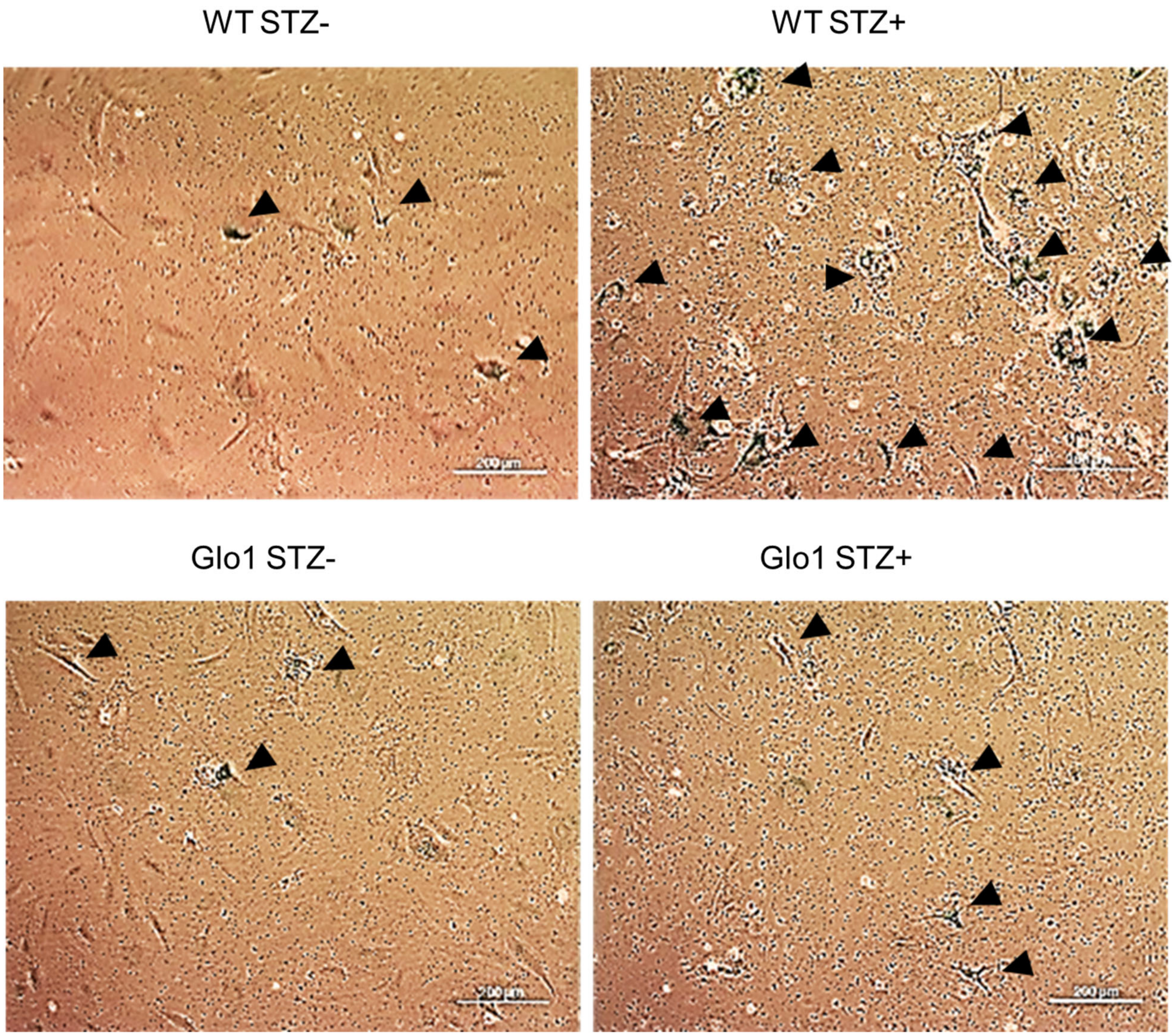

Figure S1. Measurement of cellular senescence. Representative images of β-galactosidase+ cells (arrow) within EDCs sourced from STZ treated and untreated Glo1 and WT mice. Scale bar = 200 µm.
